# Three‐dimensionally printed polycaprolactone/beta‐tricalcium phosphate scaffold was more effective as an rhBMP‐2 carrier for new bone formation than polycaprolactone alone

**DOI:** 10.1002/jbm.a.37075

**Published:** 2020-08-22

**Authors:** Su A Park, Hyo‐Jung Lee, Sung‐Yeol Kim, Keun‐Suh Kim, Deuk‐Won Jo, Shin‐Young Park

**Affiliations:** ^1^ Department of Nature‐Inspired Nanoconvergence Systems, Nanoconvergence and Manufacturing Systems Research Division Korea Institute of Machinery and Materials (KIMM) Daejeon South Korea; ^2^ Department of Periodontology, Section of Dentistry Seoul National University Bundang Hospital Seongnam‐si South Korea; ^3^ Department of Prosthodontics, Section of Dentistry Seoul National University Bundang Hospital Seongnam‐si South Korea; ^4^ Program in Dental Clinical Education and Dental Research Institute Seoul National University School of Dentistry Seoul South Korea

**Keywords:** 3D printing, beta‐tricalcium phosphate, bone regeneration, polycaprolactone, rhBMP‐2

## Abstract

Recombinant human bone morphogenetic protein 2 (rhBMP‐2) has been widely used in bone tissue engineering to enhance bone regeneration because of its osteogenic inductivity. However, clinical outcomes can vary depending on the scaffold materials used to deliver rhBMP‐2. In this study, 3D‐printed scaffolds with a ratio of 1:1 polycaprolactone and beta‐tricalcium phosphate (PCL/T50) were applied as carriers for rhBMP‐2 in mandibular bone defect models in dog models. Before in vivo application, in vitro experiments were conducted. Preosteoblast proliferation was not significantly different between scaffolds made of PCL/T50 and polycaprolactone alone (PCL/T0) regardless of rhBMP‐2 delivery. However, PCL/T50 showed an increased level of the alkaline phosphatase activity and mineralization assay when rhBMP‐2 was delivered. In in vivo, the newly formed bone volume of the PCL/T50 group was significantly increased compared with that of the PCL/T0 scaffolds regardless of rhBMP‐2 delivery. Histological examination showed that PCL/T50 with rhBMP‐2 produced significantly greater amounts of newly bone formation than PCL/T0 with rhBMP‐2. The quantities of scaffold remaining were lower in the PCL/T50 group than in the PCL/T0 group, although it was not significantly different. In conclusion, PCL/T50 scaffolds were advantageous for rhBMP‐2 delivery as well as for maintaining space for bone formation in mandibular bone defects.

## 1 INTRODUCTION

Bone morphogenetic protein 2 was first isolated by Urist^1^ and has been considered a potent growth factor to induce bone formation either orthotopically or ectopically. For bone tissue engineering, recombinant human bone morphogenetic protein 2 (rhBMP‐2) with various types of scaffolds has been the most widely investigated protein in dental and orthopedic fields.^2^, ^3^

Beta‐tricalcium phosphate (beta‐TCP) as a carrier for the clinical use of rhBMP‐2 was suggested by Urist et al. due to its capacity to stimulate the biological activity of rhBMP‐2; subsequently, a commercial prototype of rhBMP‐2 with beta‐TCP was approved by the US Food and Drug Administration in 2007.^4^, ^5^, ^6^ Bone regeneration procedures using rhBMP‐2 with beta‐TCP have been clinically performed and have resulted in successful clinical outcomes.^7^, ^8^, ^9^ Beta‐TCP in either block or particulate form is widely used for bone grafting procedures because of its osteoconductivity.^10^ In addition, beta‐TCP shows high affinity to cells and growth factors and prolongs their active time in vivo.^11^, ^12^, ^13^, ^14^, ^15^ Beta‐TCP itself, however, is fragile and brittle, which limits its ability to maintain space during the healing period, especially in significant defects in load‐bearing areas.

Recently, the development of customized 3D‐printed scaffolds has improved the ability to maintain space.^16^, ^17^ As the oral cavity is a dynamic organ that functions to chew foods and phonate, stable space maintenance is an essential requirement for scaffolds to enable successful bone regeneration.^18^, ^19^, ^20^ Various studies have attempted to fabricate polycaprolactone (PCL) scaffolds for bone regeneration in dentistry.^21^, ^22^ As PCL is both easy to handle due to its thermoplastic characteristics and biocompatible, PCL has been widely applied for use in 3D scaffold fabrication.^23^, ^24^ Although PCL scaffolds show favorable tissue responses in vivo, the scaffolds themselves have no osteogenetic potential to induce bone regeneration. Therefore, previous studies have combined PCL with beta‐TCP for the fabrication of 3D scaffolds, which have successfully improved bone healing due to the complimentary action of PCL and beta‐TCP.^25^

In this study, we delivered rhBMP‐2 to bone defects with a 3D printed scaffold system. For this purpose, a mandibular bone defect model was developed in beagles, which is a standard model in dentistry to test biomaterials for clinical application. Then, 3D‐printed scaffolds using PCL and beta‐TCP were implanted into the defects and tested as carriers for rhBMP‐2.

## 2 MATERIALS AND METHODS

### 2.1 Fabrication of scaffolds

The PCL scaffolds were designed using CAD/CAM. A 3D bioprinting system (laboratory‐made system in the Korea Institute of Machinery and Materials) was used for the construction of scaffolds. This system was equipped with a three‐axis x‐y‐z translation stage, nozzle, dispenser covered with a heating jacket to melt PCL polymer, and compression/heat controller. The scaffold was orthogonally oriented with a 400 μm lattice size. Using the 3D plotting system, two types of scaffolds were fabricated: (1) 3D scaffolds with PCL (MW 45,000; Sigma‐Aldrich, St. Louis, MO) alone (PCL/T0) and (2) 3D scaffolds with PCL and beta‐TCP (Sigma‐Aldrich, St. Louis, MO) blend (PCL/T50). For PCL/T50 scaffold fabrication, the dry PCL powder and beta‐TCP powder were mixed at a ratio of 50/50 wt % and injected into the printer dispenser barrel. For PCL/T0, the dry PCL powder alone was used. The blend in the barrel was heated at 130°C and dispensed through a nozzle with a 400 μm diameter. 3D‐printed scaffolds with dimensions of 10 x 4x 4 mm^3^ were printed layer by layer.

#### 2.1.1 *SEM examination*

SEM examination was performed for scaffold characterization. Prior to examination, scaffolds were coated with platinum under an argon atmosphere using a sputter coater (SCD 0005; BAL‐TEC, Los Angeles, CA). SEM images were taken with an accelerating voltage of 10 kV. The morphology of the scaffolds, including strand size and periods, was examined using SEM images.

### 2.2 In vitro experiments

#### 2.2.1 *Measurement of rhBMP‐2 release*

ELISA was applied to quantify the release of rhBMP‐2 from the scaffolds. Commercially available rhBMP‐2 (Novosis, CGBIO, Seongnam, Korea) was used for the experiment. Scaffolds were placed in a 24‐well plate and soaked in rhBMP‐2 at a concentration of 0.5 mg/ml in release medium (α‐MEM; Gibco) for 6 hr. The medium was collected to measure the rhBMP‐2 concentration at each time interval and frozen at −80°C for further analysis. The rhBMP‐2 concentration of each medium was measured using an ELISA kit. All experiments were triplicated.

#### 2.2.2 *Cell proliferation assay*

For the examination of cell proliferation on the scaffolds, a cell proliferation assay was performed using a 3‐(4,5‐dimethylthiazol‐2‐yl)‐2,5‐diphenyltetrazolium bromide (MTT) assay (Sigma). The mouse preosteoblast cell line MC3T3‐E1 was used in this in vitro experiment, and cells were cultured with α‐MEM (Gibco) with 10% fetal bovine serum (FBS) (Gibco) and 1% antibody. Cells were seeded at a density of 5 x 10^3^ on 96‐well plates. On days 3 and 7 of culture, MTT assay was performed and the optical density (O.D.) of each sample was measured using an automatic microplate reader (ThermoMax; Molecular Devices, Sunnyvale, CA) at a wavelength of 540 nm. The experiments were triplicated.

#### 2.2.3 *Measurement of osteogenic activity*

For measurement of the osteogenic activity of cells on the scaffolds, alkaline phosphatase (ALP) activity and mineralization assays were performed. Cells were seeded at a density of 1 x 10^4^ and cultured in osteogenic media (α‐MEM with 15% FBS supplemented with 0.2 mM ascorbic acid [Gibco] and 10 mM β‐glycerol phosphate [Gibco]). On days 7 and 14 of culture, ALP activity was measured using an ALP Activity Assay Kit (AnaSpec Inc.,Fremont., CA) and a Protein Assay Kit (iNtRON Biotechnology, Seoul, Korea). ALP activity was normalized as the concentration of p‐nitrophenol (pNP) to the protein amounts. On the day 21, mineralization analysis was performed. Fixation with 95% cold ethanol was done. Cells were stained with a 1% alizarin red S solution (Wako Chemicals, Osaka, Japan) for 5 min.

Cells were fixed in 95% cold ethanol and stained with the images of stained plates were observed. For quantitative measurement, an elution procedure with cetylpyridinium chloride in 10 mM sodium phosphate was performed and measured using an automatic microplate reader at 540 nm wavelength.

### 2.3 In vivo experiments

#### 2.3.1 *Surgical procedures*

A total of four adult dogs (male, weight 12–14 kg) with all permanent dentition were used in the in vivo experiment. Each beagle was housed in an individual cage and provided a commercial hard food diet (Dog Chow Goldpet, #35520, Cargil Argi Purnia, Inc., Pyungtaeck, Korea). We conformed to the ARRIVE guidelines for animal experiments regarding the care of animal research subjects. The study protocol was approved by the Institutional Animal Care and Use Committee of Seoul National University Bundang Hospital (IACUC No. BA1601‐192/003–01).

All the surgical procedures were performed under general anesthesia and local anesthesia. Prior to surgery, a 12‐hr fasting was kept. For the induction of the anesthesia, 5.0 mg/kg zoletil (Zoletil50, Virbac S.A., Carros, France) and 0.2 mg/kg xylazine (Rompun, Bayer Korea, Ansan, Korea) were intramuscularly injected. After endotracheal intubation, 2.2% enflurane (JW Pharmaceutical, Hwasung, Korea) and 3.0 L/min oxygen was inhaled during the surgery. For prophylaxis, an intramuscular injection of 30 mg/kg cefazolin (Chongkundang Pharm, Cheonan, Korea) was done before the surgery. At the surgical site, local anesthesia with 2% lidocaine and 1:100,000 epinephrine (Yuhan Co. Ltd., Seoul, Korea) was done.

**FIGURE 1:**
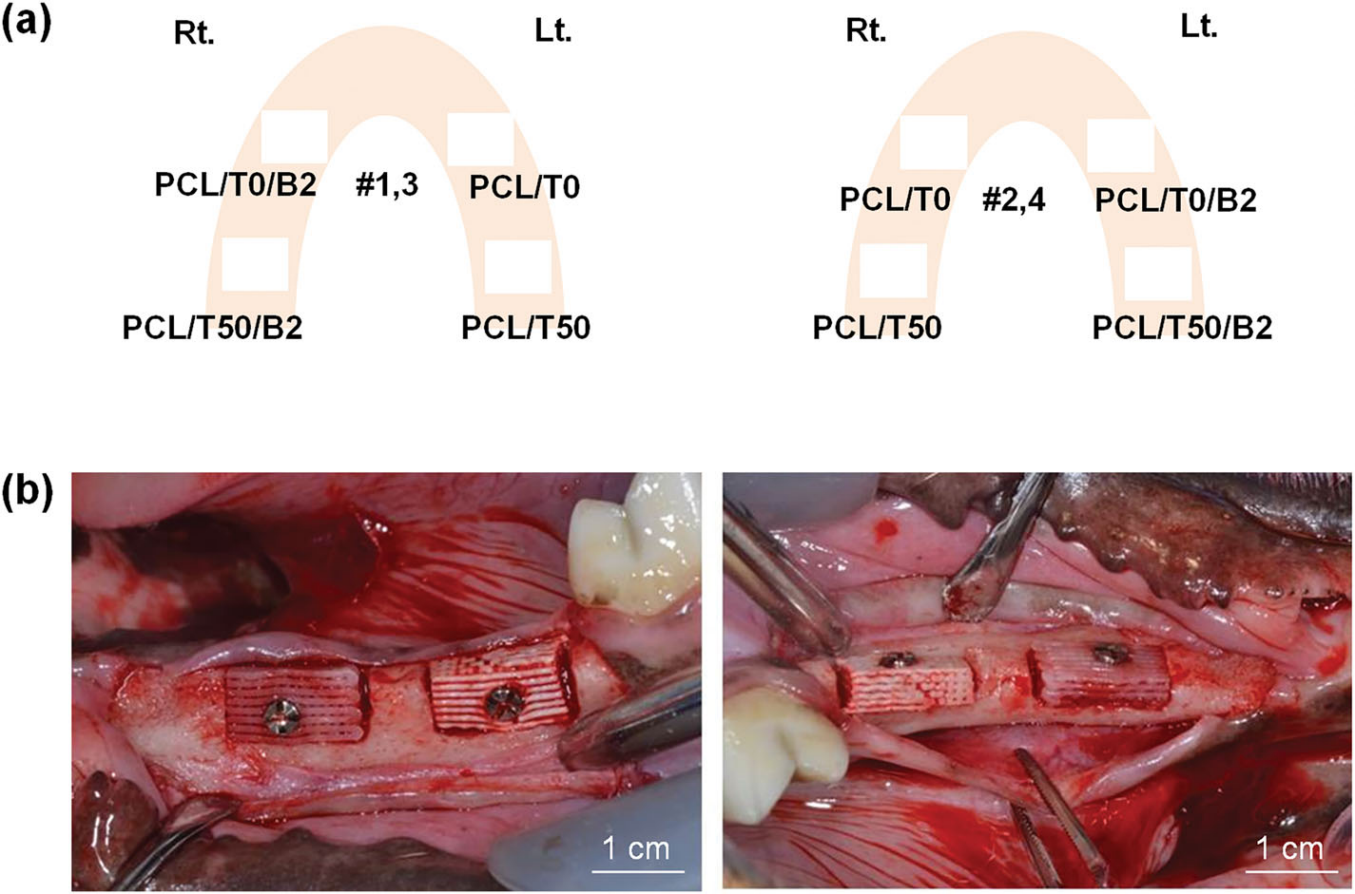
Surgical procedures for the application of scaffolds. (a) Distribution of experimental groups. (b) Clinical photographs obtained during surgery. PCL/T0, PCL alone; PCL/T50, PCL with 50% beta‐TCP; PCL/T0/B2, PCL/T0 scaffold with rhBMP‐2; PCL/T50/B2, PCL/T50 scaffold with rhBMP‐2. beta‐TCP, beta‐tricalcium phosphate; PCL, polycaprolactone; rhBMP‐2, recombinant human bone morphogenetic protein 2

After scrubbing the surgical field with a povidone‐iodine solution, both the mandibular second and third premolars were extracted in both side of animals. After 3 months of healing, surgical procedures for the implantation was performed. Crestal and vertical incisions were made in the buccal aspect of the extracted sites, and the mucoperiosteal flap was elevated. The bone defects with 10.0 × 5.0 × 5.0 mm in size^26^ were surgically created using a high‐speed engine and surgical bur under water spray. After defect formation, scaffolds were implanted into the defects. According to the scaffolds, the experimental groups were divided into four group (Figure 1): a PCL/T0 scaffold as the control, a PCL/T50 scaffold, a PCL/T0 scaffold with rhBMP‐2 (PCL/T0/B2), or a PCL/T50 scaffold with rhBMP‐2 (PCL/T50/B2). Scaffolds with rhBMP‐2 was prepared before surgery. The scaffolds were soaked in a 0.5 mg/ml rhBMP‐2 (NOVOSIS, CGBIO, Seongnam, Korea) for 6 hr before the surgery. Each scaffold was fixed with a fixation screw and covered with a resorbable membrane (Ossguide, SKbioland, Chungnam, Korea). All surgical sites underwent primary closure (Vicryl, Ethicon, Menlo Park, CA). Postoperative medications were prescribed with a single injection of 1.0 ml of dexamethasone‐21‐isonicotinate (Voren, Boehringer Ingelheim Korea Ltd., Seoul, Korea) and two injections of 1.0 ml/10.0 kg clemizole penicillin G and sodium penicillin G (Antipen‐Sm, WooGene B&G Ltd., Seoul, Korea) every other day.

All beagles were sacrificed after 3 months. After formalin perfusion of animals, block sections including the grafted sites were harvested and fixed in formaldehyde with 70% ethanol at a low temperature.

#### 2.3.2 *Micro‐computed tomography examination*

The harvested bone sections were scanned by micro‐computed tomography (micro‐CT) (SkyScan 1,173, Skyscan, Kontich, Belgium). The exposure conditions for scanning were 130 kVp and 60 mA under 1.0 mm aluminum filtration, 500 ms radiation by every 0.3° rotation, and 19.89 μm pixel size. For the measurement of the bone volume, the scanning images were reconstructed using a computer program (Nrecon Ver. 1.7.0.4, Kontich, Belgium) and analyzed using a computer program (CT‐analyzer; SkyScan, Kontich, Belgium).

#### 2.3.3 *Histology and histomorphometric analysis*

After micro‐CT scanning, fixation with 10% buffered neutral formalin (Sigma‐Aldrich Co. LLC., St. Louis) was done for 2 weeks and decalcification with formic acid (Shadon TBD‐1, Thermo Fisher Scientific Inc., Kalamazoo) was followed. The specimens were embedded in paraffin (Shadon Histocentre 3, Thermo Fisher Scientific, Inc., Kalamazoo). Then, the specimens were cut into serial sections with 3.0 μm thickness using a microtome (Shadon Finesse 325, Thermo Fisher Scientific Inc., Kalamazoo), and stained with Masson's trichrome.

For histomorphometric analysis, the newly formed bone area, scaffold area and defect area were measured using a computer program (Tomoro Scope Eye 3.5 Image Analyzer; Techsan Digital Imaging, Seoul, Korea). The region of interest (ROI) was defined as the bone defect surgically made with 5 × 5 mm.

### 2.4 Statistical analysis

Statistical analysis was performed using a computer program (STATA/SE14 software, Stata Corp, College Station, TX). For comparison of the scaffolds, student's *t* test was conducted. Less than 0.05 of *p*‐value were considered statistically significant.

## 3 RESULTS

### 3.1 Scaffold characterization

**FIGURE 2:**
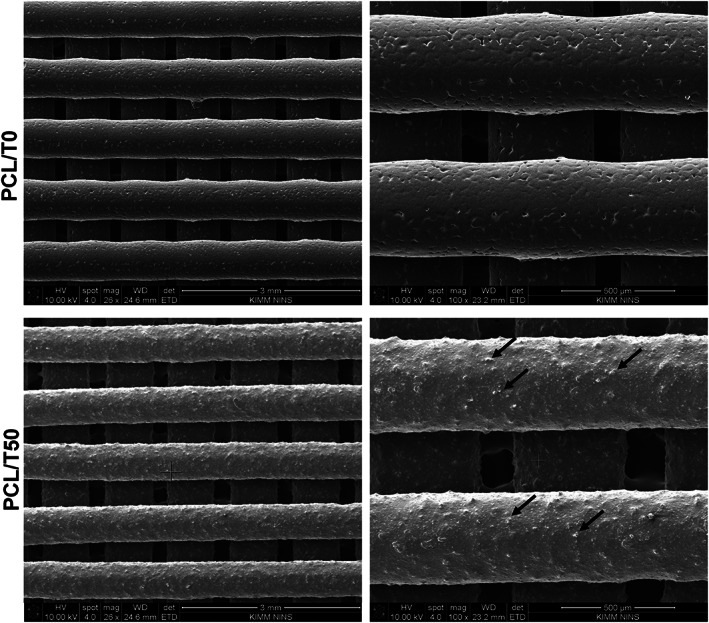
SEM images of PDL/T0 (upper) and PCL/T50 scaffolds (lower). (Arrow–beta‐TCP particles, original magnification X26 [left] and X100[right]). beta‐TCP, beta‐tricalcium phosphate; PCL, polycaprolactone

Before the experiments, the shape fidelity and morphology of the 3D‐printed PCL/T0 and PCL/T50 scaffolds were observed using SEM (Figure 2). The SEM images confirmed a well‐defined and even distribution of beta‐TCP particles within the designed scaffolds.

### 3.2 In vitro experiment

**FIGURE 3:**
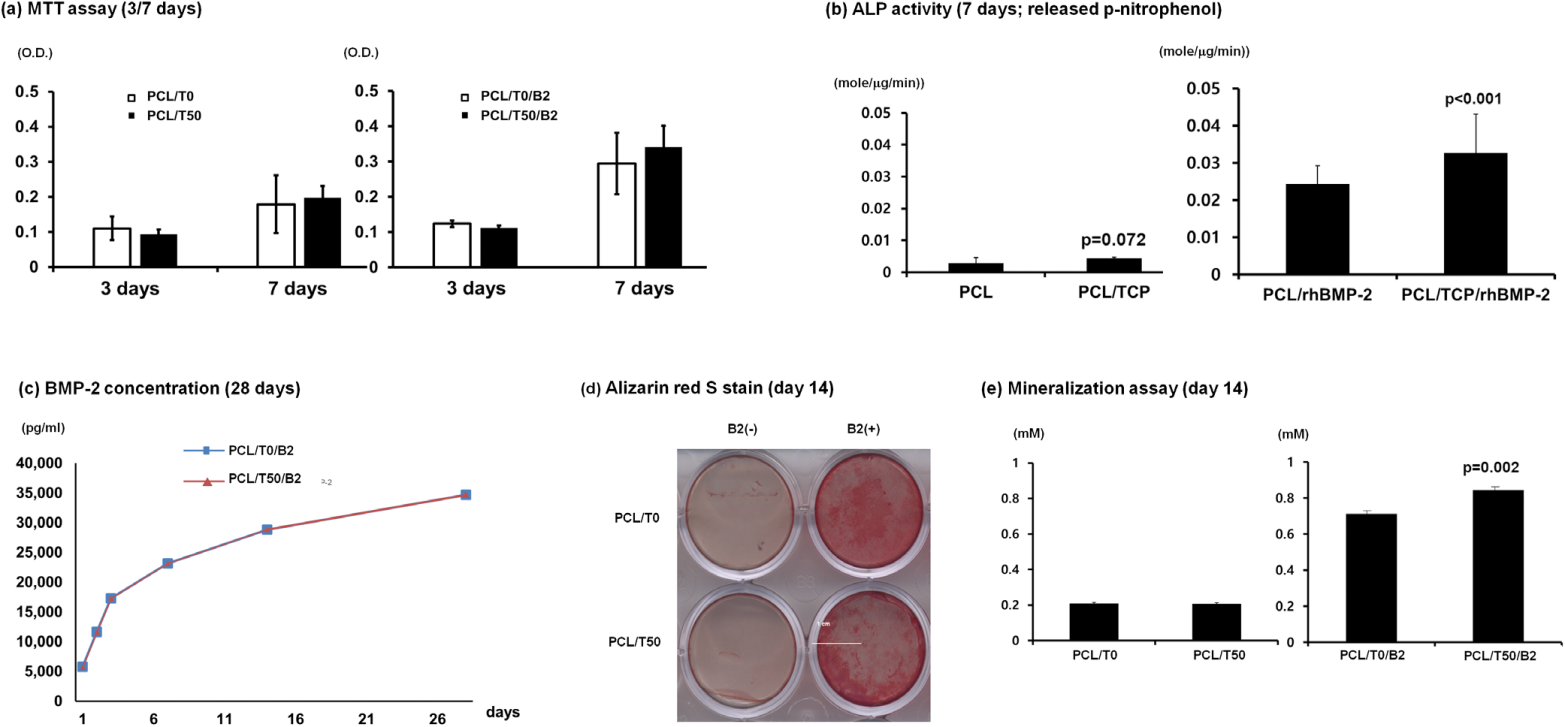
In vitro experiments using MC3T3‐E1 cells. (a) Cell proliferation (MTT) assay; (b) ALP activity; (c) Cumulative release of rhBMP‐2 from each sample for 28 days; (d) Mineralization assay for 14 days; (e) Quantification of alizarin red S‐staining. PCL/T0, PCL alone; PCL/T50, PCL with 50% beta‐TCP; PCL/T0/B2, PCL/T0 scaffold with rhBMP‐2; PCL/T50/B2, PCL/T50 scaffold with rhBMP‐2. beta‐TCP, beta‐tricalcium phosphate; MTT, 3‐(4,5‐dimethylthiazol‐2‐yl)‐2,5‐diphenyltetrazolium bromide; PCL, polycaprolactone; rhBMP‐2, recombinant human bone morphogenetic protein 2

Cell proliferation was not significantly influenced by the chemical composition of the scaffolds in either the untreated or rhBMP‐2‐treated groups (Figure 3a). The PCL/T50 scaffolds, however, significantly increased ALP activity when rhBMP‐2 was delivered (Figure 3b). The rhBMP‐2 concentration delivered by the PCL/T0 and PCL/T50 scaffolds was similar for 28 days (Figure 3c). In the mineralization assay, the PCL/T50 scaffold produced significantly more mineralization nodules (Figure 3d,e).

### 3.3 In vivo experiment

The animals healed uneventfully after the surgical procedures. After 3 months, bone regeneration was radiographically and histomorphometrically examined.

**FIGURE 4:**
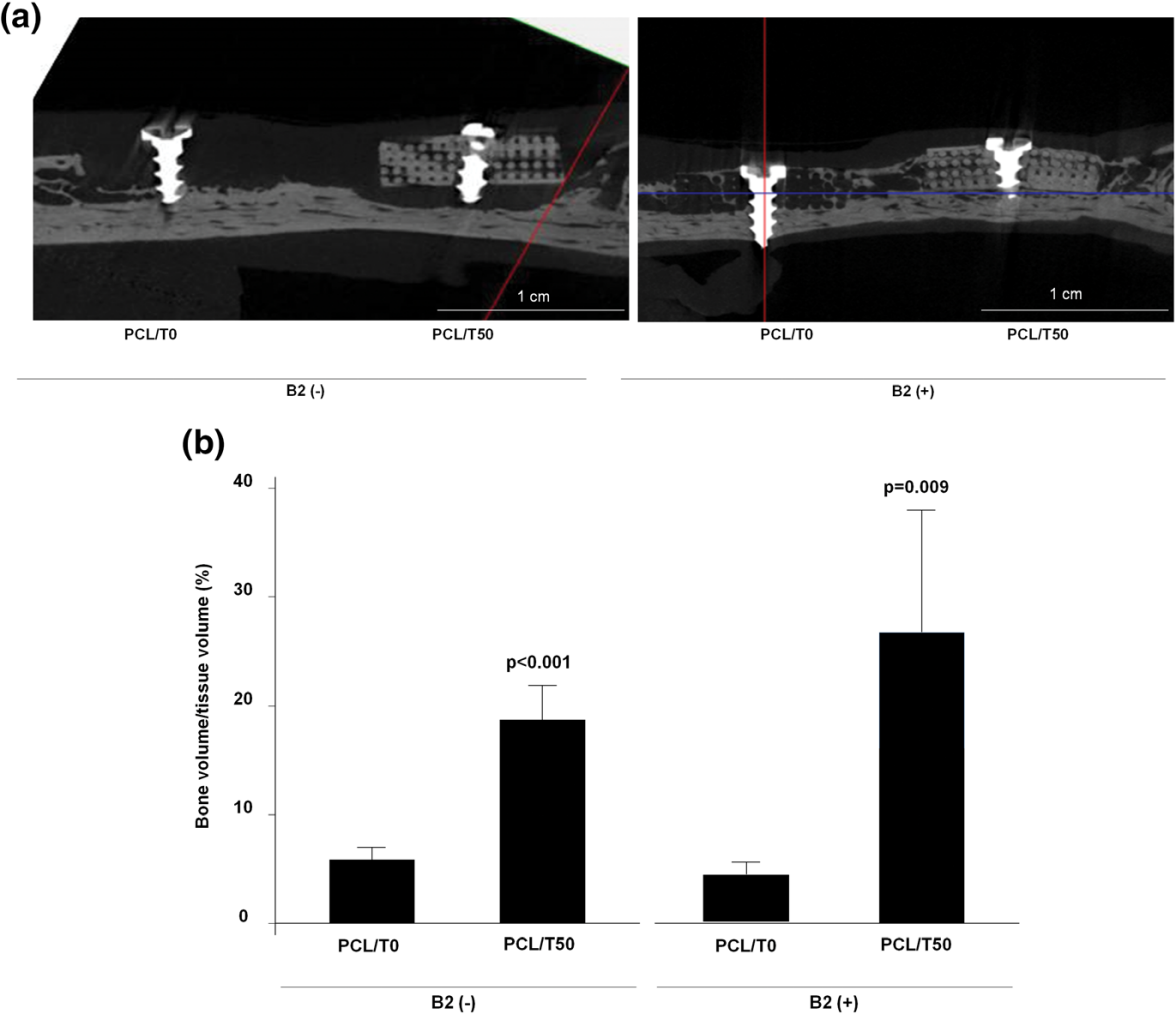
Micro‐CT images and analysis 3 months after scaffold implantation in mandibular defects in beagles. (a) Representative micro‐CT images in each group; (b) Bone volume/tissue volume (%) in each group. PCL/T0, PCL alone; PCL/T50, PCL with 50% beta‐TCP; B2, rhBMP‐2. beta‐TCP, beta‐tricalcium phosphate; Micro‐CT, micro‐computed tomography; PCL, polycaprolactone; rhBMP‐2, recombinant human bone morphogenetic protein 2

Micro‐CT examination showed that the PCL/T50 scaffolds produced significantly more new bone than the PCL/T0 scaffolds (Figure 4; *p* < .001). The PCL/T50/B2 scaffolds also induced significantly greater amounts of new bone formation than the PCL/T0/B2 scaffolds (Figure 4; *p* = .009).

**FIGURE 5:**
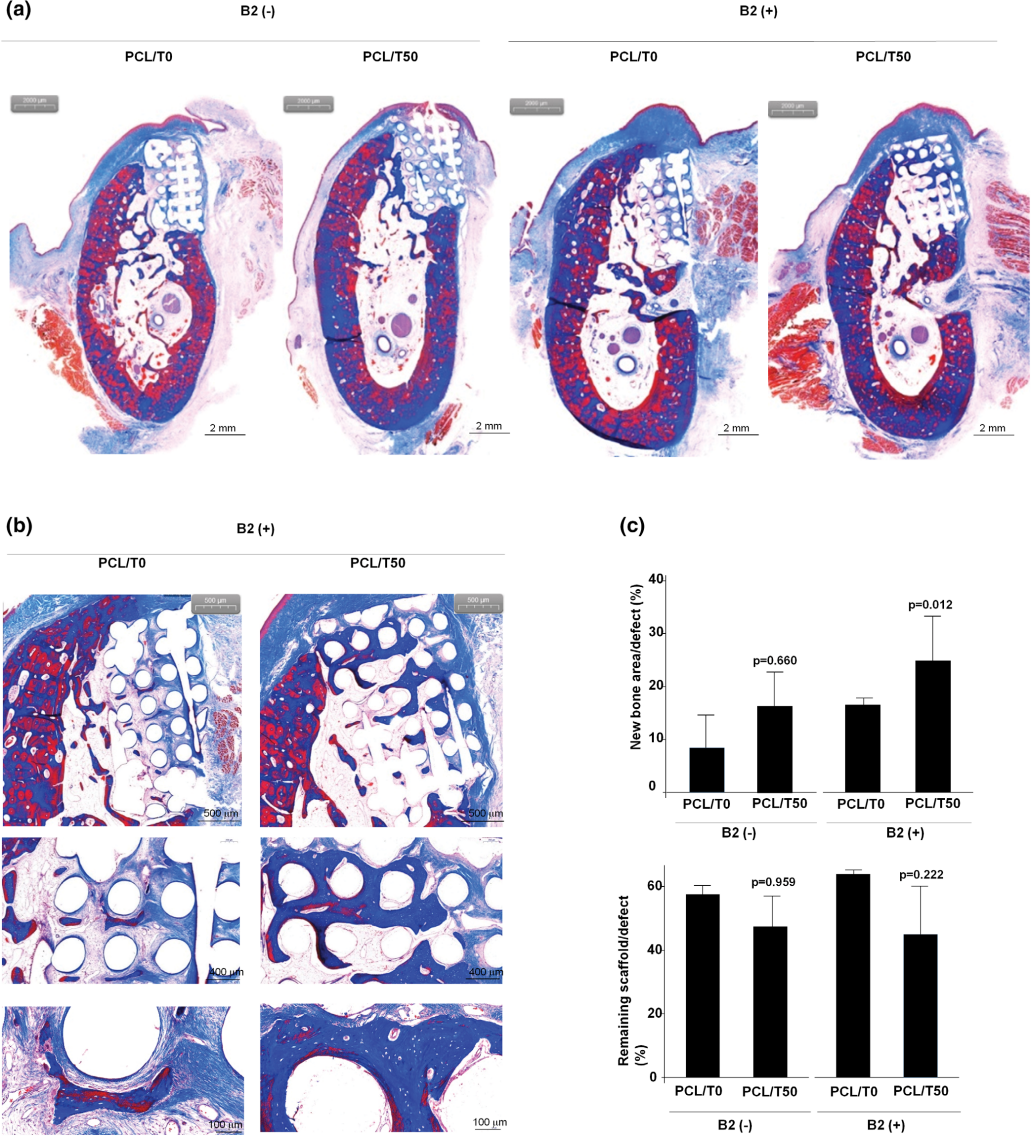
Histological images and analysis 3 months after scaffold implantation in mandibular defects in beagles. (a) Representative histological images of each group, original magnification X1.25. (b) Magnified images of PCL/T0/B2 (left) and PCL/T50 (right) scaffolds; original magnification X2 (upper) and X4 (lower). (c) New bone area/defect area ratio (left) and remaining scaffold area ratio (right) in each group. PCL/T0, PCL alone; PCL/T50, PCL with 50% beta‐TCP; B2, rhBMP‐2. beta‐TCP, beta‐tricalcium phosphate; PCL, polycaprolactone; rhBMP‐2, recombinant human bone morphogenetic protein 2

The histological examination was consistent with the micro‐CT analysis (Figure 5). The amount of new bone was the highest in the PCL/T50/B2 group. The magnified images showed that the new bone was well integrated within the scaffolds. Histomorphometric analysis also showed that the PCL/T50/B2 scaffold induced significantly greater amounts of new bone formation than the PCL/T0/B2 scaffold. In addition, less scaffold material remained in the PCL/T50 scaffold group than in the PCL scaffold group regardless of rhBMP‐2 treatment, although the difference was not significant.

## 4 DISCUSSION

In this study, the PCL/T50 scaffolds showed satisfactory results in terms of both rhBMP‐2 delivery and space maintenance in the mandibular defect model. The amount of newly formed bone induced by rhBMP‐2 was greater in the PCL/T50 group than in the PCL/T0 group with intact scaffolds.

In our previous study, we fabricated 3D scaffolds with PCL and demonstrated that 3D PCL scaffold was advantageous for space maintaining and biocompatibility in jaw bone reconstruction.^16^ Similarly, Goh et al. revealed that 3D PCL scaffold preserved vertical dimension of fresh extraction socket and allowed the normal bone healing process and implant treatment in their randomized controlled clinical trial.^21^ However, both studies failed to result in the statistically significant difference between PCL scaffolds alone and control group in bone volume.

To enhance the amounts of newly formed bone, BMP‐2 is widely applied into the defect sites in bone tissue engineering. Previously, we applied PCL scaffolds with polydopamine (DOPA) coating on the surface for rhBMP‐2 delivery.^27^ As DOPA has abundant catechol and amine groups that can be coupled with bioactive molecules containing primary amine or thiol groups, DOPA can immobilize rhBMP‐2 on the surface of PCL. As a result, the osteogenic activities of preosteoblasts were significantly increased in the PCL‐DOPA‐rhBMP‐2 group compared with the PCL‐rhBMP‐2 group. A further recent study applied bone‐forming peptide 1 (BFP1), a short peptide derived from BMP7, with PCL‐DOPA scaffolds in rabbit calvarial defects.^28^, ^29^ The DOPA coating on the scaffolds showed advantages in bone apposition on the scaffold surfaces but not in total bone volume. One of the reasons is assumed to be that a particular portion of the calvarial defect is occupied by the PCL scaffold and is not replaced by newly formed bone. That is, space for sprouting new bone may be limited when PCL scaffolds are used for bone regeneration.

In this study, mixture of PCL and beta‐TCP was utilized for 3D scaffold carrying BMP‐2. One of the merits of using beta‐TCP as a material for bone tissue regeneration is that beta‐TCP is degraded during healing and thereby allows space for bone formation.^30^ In addition, this process includes the release of a large number of calcium ions and sulfate ions to be used for new bone formation.^31^ According to the work of Dohzono et al., BMP‐2 accelerates degradation of the beta‐TCP scaffold, stimulating massive osteoclasts around the scaffolds and providing sufficient space for new bone formation.^32^ Although the poor mechanical properties of beta‐TCP have hindered the use of beta‐TCP in the load‐bearing region, mixing beta‐TCP with PCL solves this problem, as shown in this study.

Similar attempts to fabricate PCL/T50 scaffolds for bone regeneration have been reported in previous studies. Owing to the difficulty of handling beta‐TCP in the rapid prototyping type of 3D printing, most studies applied PCL with less than 20% beta‐TCP.^25^, ^33^, ^34^, ^35^, ^36^ However, low concentrations of beta‐TCP resulted in a lack of significant differences between PCL and PCL/beta‐TCP in bone regeneration.^37^ In contrast, our team previously fabricated 3D PCL/beta‐TCP scaffolds with beta‐TCP concentrations of 50 and 70%, and successful in vitro results in osteogenic differentiation were obtained.^38^ However, scaffolds with 70% beta‐TCP/PCL showed low compressive strength and were so brittle that fixation of the scaffolds into defects in the in vivo experiments was difficult. Consequently, 50% PCL/beta‐TCP was applied in this study, similar to other in vivo studies that used PCL/beta‐TCP.^39^, ^40^ Despite the high concentration of beta‐TCP used in this study, the PCL/T50 scaffolds retained structural stability after 3 months of healing, as shown in micro‐CT and histological images.

There have been several studies about a combinational approach for applying polymer/beta‐TCP/rhBMP‐2 for bone regeneration, although previous studies fabricated scaffolds containing a lower concentration of beta‐TCP than was used in this study. Abbah et al. implanted beta‐TCP/PCL (2:8) scaffolds with rhBMP‐2 into load‐bearing regions, such as lumbar interbody fusion cases, in pig models and reported complete defect bridging at 6 months.^41^ Shim et al. fabricated a barrier membrane using PCL/poly (lactic‐co‐glycolic acid) (PLGA)/beta‐TCP with an 8:2 ratio of polymer to beta‐TCP as a carrier for rhBMP‐2.^42^ They placed scaffolds over rabbit calvarial defects rather than in the defects to secure the space for bone formation. As a result, a significant amount of newly formed bone was regenerated in the defects after 4 and 8 weeks. For dental implants, Bae et al. suggested the use of a 3D‐printed beta‐TCP/PCL (3:7) scaffold with decellularized extracellular matrix and rhBMP‐2 as a surgical stent for the proper installation of dental implants as well as vertical bone regeneration.^43^

In addition to beta‐TCP, hydroxyapatite (HA) has been applied as a bone graft material in dentistry, and our team also previously produced HA/PCL scaffolds by rapid prototyping printing methods.^44^ However, as HA is a denser crystalline form of bone mineral and hardly absorbed, HA/PCL was not evaluated as a carrier for rhBMP‐2 delivery.^45^ Among previous studies, Chanchareonsook et al. fabricated HA/PCL scaffolds for rhBMP‐2 delivery in a mandibular defect model.^46^ However, scaffolds were not printed by rapid prototyping methods but fabricated by selective laser sintering methods with PCL, and HA was coated on the surface of the PCL scaffolds. HA and beta‐TCP composite materials, biphasic calcium phosphate (BCP), are also advantageous for bone regeneration because of the complementary mechanical strength of HA and the cell affinity of beta‐TCP. BCP is another possible 3D scaffold material for rhBMP‐2 delivery for evaluation in future research.

Regarding the in vivo application of rhBMP‐2, the concentration of rhBMP‐2 is critical in the occurrence of complications such as extensive swelling, immature bone formation, edema and loss of graft or implants.^6^, ^47^ Commercially available rhBMP‐2 is applied to defects at a concentration of 0.5–1 mg/ml during bone augmentation procedures. In this study, 0.5 mg/ml rhBMP‐2 was applied to the defects, and no adverse effects, such as lace‐like immature bone and loss of graft materials, were observed. Well‐matured lamellar bone was observed around the scaffolds, and the remodeling process appeared to have occurred routinely. It is thought that PCL/T50 as a carrier for rhBMP‐2 did not release a large initial burst of rhBMP‐2 into the defects but secreted rhBMP‐2 slowly, as shown in Figure 3.

## 5 CONCLUSION

Rapid prototyping for 3D scaffold fabrication is useful method for manipulation of various materials. In this study, 3D scaffolds for rhBMP‐2 is fabricated with PCL for manipulation of customized scaffolds and excellent mechanical strength and beta‐TCP for a favorable affinity for cells and proteins as well as biodegradability. As a result, PCL/T50 composite scaffolds exhibit advantages in rhBMP‐2 delivery, resulting in significant amounts of new bone formation in the bone defects.

## References

[1] Urist MR . Bone: formation by autoinduction. Science. 1965;150(3698):893‐899. 5319761 10.1126/science.150.3698.893

[2] Hutmacher DW . Scaffolds in tissue engineering bone and cartilage. Biomaterials. 2000;21(24):2529‐2543. 10.1016/S0142-9612(00)00121-6. 11071603

[3] Khan SN , Lane JM . The use of recombinant human bone morphogenetic protein‐2 (rhBMP‐2) in orthopaedic applications. Expert Opin Biol Ther. 2004;4(5):741‐748. 15155165 10.1517/14712598.4.5.741

[4] Urist MR , Nilsson O , Rasmussen J , et al. Bone regeneration under the influence of a bone morphogenetic protein (BMP) beta tricalcium phosphate (TCP) composite in skull trephine defects in dogs. Clin Orthop Relat Res. 1987;(214):295‐304. https://pubmed.ncbi.nlm.nih.gov/3791755/. 3791755

[5] Urist MR , Lietze A , Dawson E . Beta‐tricalcium phosphate delivery system for bone morphogenetic protein. Clin Orthop Relat Res. 1984;187:277‐280. 6744730

[6] Kelly MP , Vaughn OL , Anderson PA . Systematic review and meta‐analysis of recombinant human bone morphogenetic protein‐2 in localized alveolar ridge and maxillary sinus augmentation. J Oral Maxillofac Surg. 2016;74(5):928‐939. 26707429 10.1016/j.joms.2015.11.027

[7] Gomes‐Ferreira PH , Okamoto R , Ferreira S , De Oliveira D , Momesso GA , Faverani LP . Scientific evidence on the use of recombinant human bone morphogenetic protein‐2 (rhBMP‐2) in oral and maxillofacial surgery. Oral Maxillofac Surg. 2016;20(3):223‐232. 27236776 10.1007/s10006-016-0563-4

[8] Jo DW , Cho YD , Seol YJ , Lee YM , Lee HJ , Kim YK . A randomized controlled clinical trial evaluating efficacy and adverse events of different types of recombinant human bone morphogenetic protein‐2 delivery systems for alveolar ridge preservation. Clin Oral Implants Res. 2019;30(5):396‐409. 30883942 10.1111/clr.13423

[9] Zetola A , do Valle M , Littieri S , Baumgart D , Gapski R . Use of rhBMP‐2/beta‐TCP for interpositional vertical grafting augmentation: 5.5‐year follow‐up clinically and histologically. Implant Dent. 2015;24(3):349‐353. 25915407 10.1097/ID.0000000000000245

[10] Chang YL , Stanford CM , Keller JC . Calcium and phosphate supplementation promotes bone cell mineralization: implications for hydroxyapatite (HA)‐enhanced bone formation. J Biomed Mater Res. 2000;52(2):270‐278. 10951365 10.1002/1097-4636(200011)52:2<270::aid-jbm5>3.0.co;2-1

[11] Tadokoro M , Matsushima A , Kotobuki N , et al. Bone morphogenetic protein‐2 in biodegradable gelatin and beta‐tricalcium phosphate sponges enhances the in vivo bone‐forming capability of bone marrow mesenchymal stem cells. J Tissue Eng Regen Med. 2012;6(4):253‐260. 21548136 10.1002/term.427

[12] Abarrategi A , Moreno‐Vicente C , Ramos V , Aranaz I , Sanz Casado JV , Lopez‐Lacomba JL . Improvement of porous beta‐TCP scaffolds with rhBMP‐2 chitosan carrier film for bone tissue application. Tissue Eng Part A. 2008;14(8):1305‐1319. 18491953 10.1089/ten.tea.2007.0229

[13] Ohyama T , Kubo Y , Iwata H , Taki W . Beta‐tricalcium phosphate as a substitute for autograft in interbody fusion cages in the canine lumbar spine. J Neurosurg. 2002;97(suppl 3):350‐354. 12408391 10.3171/spi.2002.97.3.0350

[14] Tessmar J , M Göpferich A . Matrices and scaffolds for protein delivery in tissue engineering. Adv Drug Deliv Rev. 2007;59(4‐5):274–291. 17544542 10.1016/j.addr.2007.03.020

[15] De Witte T‐M , Fratila‐Apachitei LE , Zadpoor AA , Peppas NA . Bone tissue engineering via growth factor delivery: from scaffolds to complex matrices. Regener Biomater. 2018;5(4):197‐211. 10.1093/rb/rby013 PMC6077800 30094059

[16] Park SA , Lee HJ , Kim KS , et al. In vivo evaluation of 3D‐printed polycaprolactone scaffold implantation combined with beta‐TCP powder for alveolar bone augmentation in a beagle defect model. Materials. 2018;11(2):238. 10.3390/ma11020238 PMC5848935 29401707

[17] Hwang KS , Choi JW , Kim JH , et al. Comparative efficacies of collagen‐based 3D printed PCL/PLGA/beta‐TCP composite block bone grafts and biphasic calcium phosphate bone substitute for bone regeneration. Materials. 2017;10(4):421. 10.3390/ma10040421 PMC5506921 28772780

[18] Polo‐Corrales L , Latorre‐Esteves M , Ramirez‐Vick JE . Scaffold design for bone regeneration. J Nanosci Nanotechnol. 2014;14(1):15‐56. 24730250 10.1166/jnn.2014.9127 PMC3997175

[19] Poh PSP , Valainis D , Bhattacharya K , van Griensven M , Dondl P . Optimization of bone scaffold porosity distributions. Sci Rep. 2019;9(1):9170. 31235704 10.1038/s41598-019-44872-2 PMC6591284

[20] Bose S , Roy M , Bandyopadhyay A . Recent advances in bone tissue engineering scaffolds. Trends Biotechnol. 2012;30(10):546‐554. 22939815 10.1016/j.tibtech.2012.07.005 PMC3448860

[21] Goh BT , Teh LY , Tan DB , Zhang Z , Teoh SH . Novel 3D polycaprolactone scaffold for ridge preservation‐a pilot randomised controlled clinical trial. Clin Oral Implants Res. 2015;26(3):271‐277. 25263527 10.1111/clr.12486

[22] Rasperini G , Pilipchuk SP , Flanagan CL , et al. 3D‐printed bioresorbable scaffold for periodontal repair. J Dent Res. 2015;94(suppl 9):153S‐157S. 26124215 10.1177/0022034515588303

[23] Shim J‐H , Lee J‐S , Kim JY , Cho D‐W . Bioprinting of a mechanically enhanced three‐dimensional dual cell‐laden construct for osteochondral tissue engineering using a multi‐head tissue/organ building system. J Micromech Microeng. 2012;22(8):085014.

[24] Wei C , Cai L , Sonawane B , Wang S , Dong J . High‐precision flexible fabrication of tissue engineering scaffolds using distinct polymers. Biofabrication. 2012;4(2):025009. 22635324 10.1088/1758-5082/4/2/025009

[25] Goh BT , Chanchareonsook N , Tideman H , Teoh SH , Chow JK , Jansen JA . The use of a polycaprolactone‐tricalcium phosphate scaffold for bone regeneration of tooth socket facial wall defects and simultaneous immediate dental implant placement in *Macaca fascicularis* . J Biomed Mater Res A. 2014;102(5):1379‐1388. 23733534 10.1002/jbm.a.34817

[26] Lalone EA , Willing RT , Shannon HL , King GJ , Johnson JA . Accuracy assessment of 3D bone reconstructions using CT: an intro comparison. Med Eng Phys. 2015;37(8):729‐738. 26037323 10.1016/j.medengphy.2015.04.010

[27] Lee SJ , Lee D , Yoon TR , et al. Surface modification of 3D‐printed porous scaffolds via mussel‐inspired polydopamine and effective immobilization of rhBMP‐2 to promote osteogenic differentiation for bone tissue engineering. Acta Biomater. 2016;40:182‐191. 26868173 10.1016/j.actbio.2016.02.006

[28] Lee SJ , Won J‐E , Han C , et al. Development of a three‐dimensionally printed scaffold grafted with bone forming peptide‐1 for enhanced bone regeneration with in vitro and in vivo evaluations. J Colloid Interface Sci. 2019;539:468‐480. 30611042 10.1016/j.jcis.2018.12.097

[29] Kim HK , Kim JH , Park DS , et al. Osteogenesis induced by a bone forming peptide from the prodomain region of BMP‐7. Biomaterials. 2012;33(29):7057‐7063. 22795855 10.1016/j.biomaterials.2012.06.036

[30] Liu B , Lun DX . Current application of beta‐tricalcium phosphate composites in orthopaedics. Orthop Surg. 2012;4(3):139‐144. 22927147 10.1111/j.1757-7861.2012.00189.x PMC6583186

[31] Wang J , Chen W , Li Y , Fan S , Weng J , Zhang X . Biological evaluation of biphasic calcium phosphate ceramic vertebral laminae. Biomaterials. 1998;19(15):1387‐1392. 9758038 10.1016/s0142-9612(98)00014-3

[32] Dohzono S , Imai Y , Nakamura H , Wakitani S , Takaoka K . Successful spinal fusion by E. coli‐derived BMP‐2‐adsorbed porous beta‐TCP granules: a pilot study. Clin Orthop Relat Res. 2009;467(12):3206‐3212. 19582526 10.1007/s11999-009-0960-1 PMC2772941

[33] Khojasteh A , Behnia H , Hosseini FS , Dehghan MM , Abbasnia P , Abbas FM . The effect of PCL‐TCP scaffold loaded with mesenchymal stem cells on vertical bone augmentation in dog mandible: a preliminary report. J Biomed Mater Res B Appl Biomater. 2013;101(5):848‐854. 23359464 10.1002/jbm.b.32889

[34] Yeo A , Cheok C , Teoh SH , Zhang ZY , Buser D , Bosshardt DD . Lateral ridge augmentation using a PCL‐TCP scaffold in a clinically relevant but challenging micropig model. Clin Oral Implants Res. 2012;23(12):1322‐1332. 22145939 10.1111/j.1600-0501.2011.02366.x

[35] Park JH , Jung SY , Lee CK , et al. A 3D‐printed polycaprolactone/beta‐tricalcium phosphate mandibular prosthesis: a pilot animal study. Laryngoscope. 2019;130:358‐366. 10.1002/lary.27908. 30861134

[36] Lei Y , Rai B , Ho KH , Teoh SH . In vitro degradation of novel bioactive polycaprolactone—20% tricalcium phosphate composite scaffolds for bone engineering. Mater Sci Eng C. 2007;27(2):293‐298.

[37] Pae HC , Kang JH , Cha JK , et al. 3D‐printed polycaprolactone scaffold mixed with beta‐tricalcium phosphate as a bone regenerative material in rabbit calvarial defects. J Biomed Mater Res B Appl Biomater. 2019;107(4):1254‐1263. 30300967 10.1002/jbm.b.34218

[38] Park J , Lee SJ , Jo HH , et al. Fabrication and characterization of 3D‐printed bone‐like β‐tricalcium phosphate/polycaprolactone scaffolds for dental tissue engineering. J Ind Eng Chem. 2017;46:175‐181.

[39] Konopnicki S , Sharaf B , Resnick C , et al. Tissue‐engineered bone with 3‐dimensionally printed beta‐tricalcium phosphate and polycaprolactone scaffolds and early implantation: an in vivo pilot study in a porcine mandible model. J Oral Maxillofac Surg. 2015;73(5):1016 e1‐1016 e11. 25883004 10.1016/j.joms.2015.01.021

[40] Sharaf B , Faris CB , Abukawa H , et al. Three‐dimensionally printed polycaprolactone and beta‐tricalcium phosphate scaffolds for bone tissue engineering: an in vitro study. J Oral Maxillofac Surg. 2012;70(3):647‐656. 22079064 10.1016/j.joms.2011.07.029

[41] Abbah SA , Lam CX , Hutmacher DW , Goh JC , Wong HK . Biological performance of a polycaprolactone‐based scaffold used as fusion cage device in a large animal model of spinal reconstructive surgery. Biomaterials. 2009;30(28):5086‐5093. 19540586 10.1016/j.biomaterials.2009.05.067

[42] Shim JH , Yoon MC , Jeong CM , et al. Efficacy of rhBMP‐2 loaded PCL/PLGA/beta‐TCP guided bone regeneration membrane fabricated by 3D printing technology for reconstruction of calvaria defects in rabbit. Biomed Mater. 2014;9(6):065006. 25384105 10.1088/1748-6041/9/6/065006

[43] Bae JC , Lee JJ , Shim JH , et al. Development and assessment of a 3D‐printed scaffold with rhBMP‐2 for an implant surgical guide stent and bone graft material: a pilot animal study. Materials. 2017;10(12). 10.3390/ma10121434. PMC5744369 29258172

[44] Park SA , Lee SH , Kim WD . Fabrication of porous polycaprolactone/hydroxyapatite (PCL/HA) blend scaffolds using a 3D plotting system for bone tissue engineering. Bioprocess Biosyst Eng. 2011;34(4):505‐513. 21170553 10.1007/s00449-010-0499-2

[45] Hollister SJ . Porous scaffold design for tissue engineering. Nat Mater. 2005;4(7):518‐524. 16003400 10.1038/nmat1421

[46] Chanchareonsook N , Tideman H , Feinberg SE , et al. Segmental mandibular bone reconstruction with a carbonate‐substituted hydroxyapatite‐coated modular endoprosthetic poly(ɛ‐caprolactone) scaffold in *Macaca fascicularis* . J Biomed Mater Res B Appl Biomater. 2014;102(5):962‐976. 24259321 10.1002/jbm.b.33077

[47] Wikesjö U , Huang Y‐H , Xiropaidis A , et al. Bone formation at rhBMP‐2 coated titanium implants in the posterior maxilla (type IV bone) in nonhuman primates. J Clin Periodontol. 2008;35:992‐1000. 18976396 10.1111/j.1600-051X.2008.01322.x

